# S113. THE ASSOCIATION BETWEEN SCHIZOTYPAL COMPONENTS AND CONSPIRACIST BELIEFS THROUGH COGNITIVE MEDIATORS

**DOI:** 10.1093/schbul/sby018.900

**Published:** 2018-04-01

**Authors:** David Barron, Adrian Furnham, Laura Weis, Kevin Morgan, Tony Towell, Viren Swami

**Affiliations:** 1 Perdana University; 2 University College London; 3 University of Westminster; 4 Anglia Ruskin University

## Abstract

**Background:**

Belief in conspiracy theories (i.e., a subset of false narratives in which the ultimate cause of an event is believed to be due to a malevolent plot by multiple actors working together) is a widespread and stable aspect of contemporary public opinion. Given such findings, researchers have sought to understand the factors that make someone more or less likely to adopt conspiracist beliefs. More specifically, scholars have focused primarily on social and differential aspects, as well as possible psychopathological elements. These endeavours have led to reports of significant associations between schizotypal facets (odd or magical thinking and, to a lesser extent, ideas of references) and the endorsement of conspiracist beliefs. However, one limitation of extant findings is the assumption that the afore-mentioned relationships are ultimately direct; that is, schizotypal facets are directly associated with conspiracist beliefs, rather than influenced by mediating processes. To overcome this limitation, the present study sought to replicate previous findings by confirming the relationships between components of schizotypy and conspiracist beliefs. Second, this study examined the mediating influence of cognitive processes on this relationship.

**Methods:**

An international online sample of 411 women and men completed measures of schizotypal components (i.e., odd beliefs or magical thinking and ideas of reference), conspiracist beliefs, and cognitive processes (i.e., need for cognition, analytic thinking, and cognitive insight).

**Results:**

Through path analysis, results indicated associations between both schizotypal facets and conspiracist beliefs in the present sample. Further, there was evidence for the association between analytic thinking and conspiracist beliefs, and between cognitive insight and conspiracist beliefs. Indeed, cognitive insight was found to mediate the association between odd beliefs or magical thinking and ideas of reference with conspiracist beliefs. In addition, analytic thinking provided a mediating link to conspiracy ideation for odd beliefs or magical thinking, this was not found with ideas of reference. Despite an association between odd beliefs or magical thinking and need for cognition, this did not extend to conspiracist beliefs.

**Discussion:**

In summary, the results of this study supported the association between schizotypal components and conspiracist beliefs. However, they also extend previous research by suggesting that cognitive processes mediate this link. That is, although a direct link between these variables may be tenable, it is also important to consider the possible ways in which schizotypy influences cognitive processes, which in turn have an effect on conspiracist beliefs. From a practical point-of-view, this highlights possible intervention routes for reducing conspiracist beliefs, either by targeting schizotypal traits indirectly or cognitive factors directly. While this research addresses schizotypy, patients with psychotic disorders and those with an at-risk mental state have also been shown to have reasoning biases. Therefore, future research, in relation to the clinical spectrum, should consider not only reasoning biases, but an outcome of conspiracy beliefs.

